# Using Interpretable Machine Learning to Identify Baseline Predictive Factors of Remission and Drug Durability in Crohn’s Disease Patients on Ustekinumab

**DOI:** 10.3390/jcm11154518

**Published:** 2022-08-03

**Authors:** María Chaparro, Iria Baston-Rey, Estela Fernández Salgado, Javier González García, Laura Ramos, María Teresa Diz-Lois Palomares, Federico Argüelles-Arias, Eva Iglesias Flores, Mercedes Cabello, Saioa Rubio Iturria, Andrea Núñez Ortiz, Mara Charro, Daniel Ginard, Carmen Dueñas Sadornil, Olga Merino Ochoa, David Busquets, Eduardo Iyo, Ana Gutiérrez Casbas, Patricia Ramírez de la Piscina, Marta Maia Boscá-Watts, Maite Arroyo, María José García, Esther Hinojosa, Jordi Gordillo, Pilar Martínez Montiel, Benito Velayos Jiménez, Cristina Quílez Ivorra, Juan María Vázquez Morón, José María Huguet, Yago González-Lama, Ana Isabel Muñagorri Santos, Víctor Manuel Amo, María Dolores Martín Arranz, Fernando Bermejo, Jesús Martínez Cadilla, Cristina Rubín de Célix, Paola Fradejas Salazar, Antonio López San Román, Nuria Jiménez, Santiago García-López, Anna Figuerola, Itxaso Jiménez, Francisco José Martínez Cerezo, Carlos Taxonera, Pilar Varela, Ruth de Francisco, David Monfort, Gema Molina Arriero, Alejandro Hernández-Camba, Francisco Javier García Alonso, Manuel Van Domselaar, Ramón Pajares-Villarroya, Alejandro Núñez, Francisco Rodríguez Moranta, Ignacio Marín-Jiménez, Virginia Robles Alonso, María del Mar Martín Rodríguez, Patricia Camo-Monterde, Iván García Tercero, Mercedes Navarro-Llavat, Lara Arias García, Daniel Hervías Cruz, Sebastian Kloss, Alun Passey, Cynthia Novella, Eugenia Vispo, Manuel Barreiro-de Acosta, Javier P. Gisbert

**Affiliations:** 1 Hospital Universitario de La Princesa, Instituto de Investigación Sanitaria Princesa (IIS-IP), Universidad Autónoma de Madrid, 28006 Madrid, Spain; mariachs2005@gmail.com (M.C.); cristina.rubin.92@hotmail.com (C.R.d.C.); javier.p.gisbert@gmail.com (J.P.G.); 2 Centro de Investigación Biomédica en Red de Enfermedades Hepáticas y Digestivas (CIBERehd), 28029 Madrid, Spain; 3 Complejo Hospitalario Universitario de Santiago, 15706 Santiago de Compostela, Spain; iria.baston@gmail.com (I.B.-R.); manubarreiro@hotmail.com (M.B.-d.A.); 4 Complejo Hospitalario de Pontevedra, 36164 Pontevedra, Spain; estela.fernandez.salgado@sergas.es; 5 Hospital Público Comarcal la Inmaculada, 04600 Almería, Spain; jagoga0031@gmail.com; 6 Hospital Universitario de Canarias, 38320 Tenerife, Spain; laura7ramos@gmail.com; 7 Hospital Universitario A Coruña, 15006 A Coruña, Spain; maitedlp@yahoo.es; 8 Hospital Universitario Virgen Macarena, 41009 Seville, Spain; farguelles@telefonica.net; 9 Facultad de Medicina, Universidad de Sevilla, 41009 Seville, Spain; 10 Hospital Universitario Reina Sofía, 14004 Córdoba, Spain; evaiflores@gmail.com; 11 Hospital Universitario Virgen de Valme, 41014 Seville, Spain; mercedes.cabello41@gmail.com; 12 Complejo Hospitalario de Navarra, 31008 Pamplona, Spain; saioa.rubio.iturria@cfnavarra.es; 13 Hospital Universitario Virgen del Rocío, 41013 Seville, Spain; andreanuor@gmail.com; 14 Hospital de Barbastro, 22300 Barbastro, Spain; dracharro@gmail.com; 15 Hospital Universitario Son Espases, 07120 Palma, Spain; daniel.ginard@gmail.com; 16 Hospital San Pedro de Alcántara, 10003 Cáceres, Spain; cdsadornil@gmail.com; 17 Hospital Universitario Cruces, 48903 Barakaldo, Spain; olga.merino@euskalnet.net; 18 Hospital Universitari de Girona Doctor Josep Trueta, 17007 Girona, Spain; dbusquets.girona.ics@gencat.cat; 19 Hospital Comarcal de Inca, 07300 Inca, Spain; eduiyo20@hotmail.com; 20 Hospital General Universitario de Alicante, Instituto de Investigación Sanitaria y Biomédica de Alicante (ISABIAL), 03010 Alicante, Spain; gutierrez_anacas@gva.es; 21 Hospital Universitario de Araba-Txagorritxu, 01004 Vitoria-Gasteiz, Spain; patri_rami@hotmail.com; 22 Hospital Clínico Universitario de Valencia, 46010 Valencia, Spain; maiabosca@yahoo.es; 23 Hospital Clínico Universitario Lozano Blesa, 50009 Zaragoza, Spain; tarroyo.salud.aragon@gmail.com; 24 Hospital Universitario Marqués de Valdecilla, IDIVAL, 39008 Santander, Spain; garcia_maria86@hotmail.com; 25 Hospital de Manises, 46940 Manises, Spain; hinova200@gmail.com; 26 Hospital de la Santa Creu i Sant Pau, 08041 Barcelona, Spain; jordigabalos@hotmail.com; 27 Hospital Universitario 12 de Octubre, 28041 Madrid, Spain; pilarmarmon123@gmail.com; 28 Hospital Clínico Universitario de Valladolid, 47003 Valladolid, Spain; benitovelayos@hotmail.com; 29 Hospital Marina Baixa, 03570 Villajoyosa, Spain; agherola@gmail.com; 30 Hospital Universitario Juan Ramón Jiménez, 21002 Huelva, Spain; juanma_cartaya@hotmail.com; 31 Hospital General Universitario de Valencia, 46014 Valencia, Spain; josemahuguet@gmail.com; 32 Hospital Universitario Puerta de Hierro, 28222 Majadahonda, Spain; ygonzalezlama@gmail.com; 33 Hospital Universitario de Donostia, 20014 Donostia-San Sebastián, Spain; anaisabel.munagorrisantos@osakidetza.eus; 34 Hospital Regional de Málaga, 29010 Málaga, Spain; vatdig@gmail.com; 35 Hospital Universitario de La Paz, 28046 Madrid, Spain; mmartinarranz@salud.madrid.org; 36 Instituto de Investigación Sanitaria del Hospital La Paz (IdiPaz), 28046 Madrid, Spain; fbermejos1@gmail.com; 37 Hospital Universitario de Fuenlabrada, 28942 Fuenlabrada, Spain; 38 Hospital Alvaro Cunqueiro, 36213 Vigo, Spain; jesus.martinez.cadilla@sergas.es; 39 Hospital Virgen de la Concha, 49022 Zamora, Spain; paofradejas@msn.com; 40 Hospital Universitario Ramón y Cajal, 28034 Madrid, Spain; alsanroman@salud.madrid.org; 41 Hospital General Universitario de Elche, 03203 Elche, Spain; nurjime@gmail.com; 42 Hospital Universitario Miguel Servet, 50009 Zaragoza, Spain; sgarcia.lopez@gmail.com; 43 Hospital General Universitario de Castellón, 12004 Castellón de la Plana, Spain; anna_22fs@hotmail.com; 44 Hospital Universitario de Galdakao-Usansolo, 48960 Galdakao, Spain; itxasoj@gmail.com; 45 Hospital Universitario Sant Joan de Reus, 43204 Reus, Spain; fjmartinez@grupsagessa.com; 46 Hospital Clínico Universitario San Carlos, Instituto de Investigación del Hospital Clínico San Carlos [IdISSC], 28040 Madrid, Spain; carlos.taxonera@salud.madrid.org; 47 Hospital Universitario de Cabueñes, 33203 Gijón, Spain; trastoy@hotmail.com; 48 Hospital Universitario Central de Asturias, Instituto de Investigación Biosanitaria del Principado de Asturias [ISPA], 33011 Oviedo, Spain; ruthdefrancisco@gmail.com; 49 Consorci Sanitari de Terrassa, 08227 Terrassa, Spain; dmonfort@cst.cat; 50 Complejo Hospitalario Universitario de Ferrol, 15405 Ferrol, Spain; gema.molina.arriero@sergas.es; 51 Hospital Universitario Nuestra Señora de Candelaria, 38010 Santa Cruz de Tenerife, Spain; dr.alejandrohc@gmail.com; 52 Hospital Universitario Rio Hortega, 47012 Valladolid, Spain; fj.garcia.alonso@gmail.com; 53 Hospital Universitario de Torrejón, 28850 Torrejón de Ardoz, Spain; manuelvandomselaar@yahoo.com; 54 Hospital Universitario Infanta Sofía, 28702 San Sebastián de los Reyes, Spain; ramon.pajares@salud.madrid.org; 55 Hospital Clínico Universitario de Salamanca, 37007 Salamanca, Spain; alnual@saludcastillayleon.es; 56 Hospital Universitario Bellvitge, 08907 L’Hospitalet de Llobregat, Spain; frmoranta@bellvitgehospital.cat; 57 Hospital Universitario Gregorio Marañón, 28009 Madrid, Spain; drnachomarin@hotmail.com; 58 Hospital Universitario Vall d’Hebrón, 08035 Barcelona, Spain; virgiroblesalonso@gmail.com; 59 Hospital Universitario Virgen de las Nieves, 18014 Granada, Spain; mariadelmarmar@hotmail.com; 60 Hospital Universitario San Jorge, 22004 Huesca, Spain; patriciacamo@hotmail.com; 61 Hospital General Universitario Santa Lucía, 30202 Cartagena, Spain; iagtsota@hotmail.com; 62 Hospital de Sant Joan Despí Moisès Broggi, 08970 Sant Joan Despí, Spain; mnavarrollavat@gmail.com; 63 Hospital Universitario de Burgos, 09006 Burgos, Spain; laradigest@yahoo.es; 64 Hospital General Universitario de Ciudad Real, 13005 Ciudad Real, Spain; danielhervias@gmail.com; 65 Janssen, EMEA, 2340 Beerse, Belgium; skloss@its.jnj.com (S.K.); apassey@its.jnj.com (A.P.); 66 Janssen Medical Department, Paseo Doce Estrellas, 5-7, 28042 Madrid, Spain; mvispobu@its.jnj.com

**Keywords:** Crohn’s Disease, ustekinumab, predictive factors

## Abstract

Ustekinumab has shown efficacy in Crohn’s Disease (CD) patients. To identify patient profiles of those who benefit the most from this treatment would help to position this drug in the therapeutic paradigm of CD and generate hypotheses for future trials. The objective of this analysis was to determine whether baseline patient characteristics are predictive of remission and the drug durability of ustekinumab, and whether its positioning with respect to prior use of biologics has a significant effect after correcting for disease severity and phenotype at baseline using interpretable machine learning. Patients’ data from SUSTAIN, a retrospective multicenter single-arm cohort study, were used. Disease phenotype, baseline laboratory data, and prior treatment characteristics were documented. Clinical remission was defined as the Harvey Bradshaw Index ≤ 4 and was tracked longitudinally. Drug durability was defined as the time until a patient discontinued treatment. A total of 439 participants from 60 centers were included and a total of 20 baseline covariates considered. Less exposure to previous biologics had a positive effect on remission, even after controlling for baseline disease severity using a non-linear, additive, multivariable model. Additionally, age, body mass index, and fecal calprotectin at baseline were found to be statistically significant as independent negative risk factors for both remission and drug survival, with further risk factors identified for remission.

## 1. Introduction

Crohn’s Disease (CD) is a chronic inflammatory bowel disease that can result in ulceration, thickening of the intestinal wall, and a broad array of symptoms and complications. The immune mechanisms underlying CD are becoming better understood, and therefore more therapeutic options have appeared in recent years, including use of immunomodulators and biologic agents. Ustekinumab is a drug treatment in the latter category, targeting interleukin (IL)-12 and IL-23, that is indicated for the treatment of adult patients with moderately to severely active CD who have had an inadequate response with, lost response to, or were intolerant to either conventional therapy or a TNFα antagonist or have medical contraindications to such therapies [1]. The positioning of UST in particular versus anti-integrin medications is a topic of active discussion in the clinical literature [2,3].

The use of biomarkers, clinical data, and prediction models holds promise of finding the best drug for the right patient at the right time. Although clinical trials offer the gold standard by way of direct comparison between alternative treatments, they can also have certain limitations based on their generalizability (restrictive eligibility criteria, strict designs, and commonly binary endpoints). On the contrary, real-world studies and pragmatic observational trials can better reflect clinical practice and represent a greater diversity of patients, including ethnic background, biologic constituency, geographic representation, with sometimes longer follow ups and bigger cohort sizes, and hence are an important supplementary source of information to clinical trials. Real-world data from electronic medical records and claims data often come with very large sample sizes but are also constrained by data quality issues. In contrast, observational studies such as SUSTAIN [4] benefit from high-quality data capture and a pragmatic view of clinical practice. The objective of the current study was to determine whether baseline patient characteristics, including prior treatment history, are predictive of remission and drug durability after controlling for disease severity at baseline.

## 2. Materials and Methods

**Figure 1:**
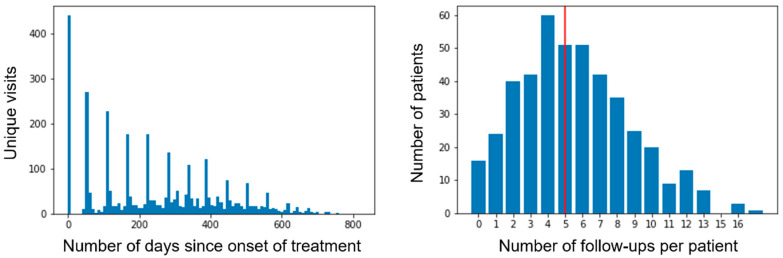
Number of unique visits over time (**left**) and number of follow-up visits per patient; red line indicates the mean number of visits (**right**).

The SUSTAIN study [4] is a retrospective multicenter study comprising 463 patients on ustekinumab (UST) with active CD (Harvey-Bradshaw Index (HBI) > 4). The HBI index was recorded by gastroenterologists in the follow-up of patients. HBI measurement was not available for 24 patients, who were excluded from the analysis, leaving a cohort size of 439 from 61 centers. Patients were evaluated at baseline and then during the follow-up for an average of 5.5 visits (median 5), and an average frequency of every 8 weeks, generating a total of 2876 visits in total where HBI was recorded (Figure 1). This creates an opportunity for longitudinal analysis as well as drug durability, which are the focus of this study.

The patient cohort exhibited a median HBI of 8.0 at baseline, with just under 50% with mild disease (5 ≤ HBI < 7), 45% with moderate disease (7 ≤ HBI ≤ 16), and 5% with severe disease (HBI > 16). The cohort was refractory, defined as having failed to respond to or remain on at least one other treatment line in the past, with 96.1% of the cohort being anti-TNF experienced and 23.7% also anti-integrin experienced prior to the onset of treatment with UST. A total of 60% had had at least one surgery; at baseline, 28% were treated with concomitant steroids and 30% with immunosuppressants (65% azathioprine, 28.8% methotrexate, and 8% Mercaptopurine).

The analysis was primarily focused on remission as a dichotomous endpoint, achieving or not remission, defined as a HBI value of ≤ 4. Importantly, the analysis was longitudinal and considered all available follow-ups per patient, rather than focusing on a specific cross-section, with temporal dependence handled by adding “days since onset of treatment” as an independent variable. Drop-out bias was handled using the last-value-carried-forward (LVCF) imputation as detailed in Appendix A.

As a secondary endpoint, drug durability was considered, defined as the time to drop-out, which is right-censored by the last follow-up. In addition to its clinical significance, drug durability served to reinforce findings that replicate across both analyses, given its lack of sensitivity to drop-out bias.

A complete table of all features used can be found in Table 1. These included 20 baseline features, time as a longitudinal covariate, and the number of concomitant steroids courses administered during the treatment window. Both models (remission and durability) were able to handle both discrete and numeric variables. For interpretability reasons, all numeric variables, except *days since the start of UST treatment*, were discretized using a model-driven technique that automatically determines the cut-offs that are optimal for the given multivariable analysis (see Appendix A). For example, the model-driven lower cut-off for the Body-Mass-Index (BMI) is 27, rather than the clinical threshold of 17. The groups employed for discretization are shown in the fourth column of Table 1. Following discretization, a separate model of remission was built on discretized variables only. In particular, the *time elapsed between diagnosis and start of treatment*, the *age at the time of consent*, *BMI*, and laboratory tests (*baseline* *albumin, baseline fecal calprotectin, baseline haemoglobin,* and *baseline CRP*), *the number of surgeries prior to onset of treatment*, the *number of comorbidities*, and the *number of anti-TNF episodes*, and similarly with the *number of anti-integrin episodes* were used as raw numeric values initially and discretized for interpretability. Treatment episodes refer to the number of times a patient has been treated with the respective drug class. So, for example, a patient with 2 treatment episodes on anti-integrin would have been started and stopped anti-integrin treatment twice, but in both cases, using vedolizumab, which is the only anti-integrin used in this cohort. Alternative codifications of exposure to anti-TNF or anti-integrin were tried out, including *total duration of treatment* (number of days on drug) and *exposure* (yes/no). All had a similar effect directionally on the model, but *number of treatment episodes* offered the best fit overall.

Baseline measurements were used for two reasons: to control for disease severity at the onset of treatment, but also as predictors of remission given information that is available at a clinically actionable point and could in the future inform a decision to treat by the clinician.

*HBI at baseline* was the only numeric variable that was discretized pre-modelling into mild, moderate, and severe disease using the clinical definition described earlier, to facilitate investigation of its interactions with other variables. In particular, a large percentage of patients were under either steroids or immunosuppressants (or both) at the study onset, which might have masked what would have otherwise been a higher HBI value. These variables were considered as having interaction effects with baseline HBI.

*BMI at baseline* was estimated using height and weight measurements where possible and inferred using regression imputation otherwise. *Sex* was also considered. Given the wide range of different *comorbidities*, these were not considered individually but rather a single variable was introduced counting the number of comorbidities present.

Disease type was described by the following variables: whether the patient had *perianal disease*, *CD* *location* according to the Montreal classification, ileal (L1), colonic (L2), or ileocolonic (L3), whether the *upper gastrointestinal tract (L4) was involved*, and whether there was *ever an extraintestinal manifestation*. *CD location* was collapsed into a dichotomous variable capturing whether the ileum was involved (Yes/No), as it was observed that there was no statistically significant difference between the effect of ileocolonic (L3) vs. ileal only (L1). Additionally, a *family history of CD* was also encoded as a dichotomous variable.

The patient journey was a semi-structured data source that can be summarized in a tabular form in a variety of ways. This analysis considered the *number of surgeries before UST treatment onset*, discretized into groups for 0, 1–2, and 3+, and treated like an ordinal factor. Previous biologic use was a key object of interest for the study. It was encoded as the *number of anti-TNF episodes* and, separately, the *number anti-integrin episodes.* Sensitivity analyses were conducted with alternative encodings including the cumulative length of treatment episodes measured in days, or measured as a proportion of time since diagnosis, and maintained a similar direction and significance levels.

The *number of concomitant steroid courses* that were given concomitantly with UST treatment was also considered in the model as a control, to improve the generalizability of the findings and attempt to extrapolate on the possibility of steroid-free remission.

Three separate analyses were run, namely, longitudinal remission non-discretized, longitudinal remission discretized, and drug durability. The primary analysis was a longitudinal remission analysis that measured the effect of different covariates on the probability of remission at different times since onset of treatment. This model was fitted on continuous variables first and then on discretized versions for interpretability reasons. The secondary analysis was a drug durability analysis focused on the time each patient stayed on ustekinumab. In the longitudinal analysis, every patient follow-up constituted a single observation, and the set of covariates included the *time since onset of treatment*, allowing for follow-ups made at different times to become comparable by controlling for the effect of time, while at the same time extracting an explicit representation of a non-linear trajectory of the endpoint over the duration of the treatment. Such use of time as a predictor notably increases the effective sample size of the analysis. Baseline covariates featured identical values for all observations coming from the same patient. LVCF techniques were used to correct for possible drop-out bias (see Appendix A). Given a dichotomous dependent variable, we were able to assess goodness-of-fit by evaluating R2 (proportion of variance explained) as well as a standard metric of classification accuracy, Area Under the Curve (AUC), using 10-fold cross validation, treating the remission model as a classifier. Drug durability featured instead one observation per patient and hence had less power than the longitudinal analysis but benefitted from a lack of sensitivity to drop-out bias.

All three analyses relied on the Generalized Additive Model (GAM) framework, introduced [5] as an extension of linear and logistic regression that allows for data-driven discovery of non-linear effects between each predictor and the remission flag using splines, while preserving its interpretable properties such as *p*-values and confidence intervals on additive effects. Survival analysis is a recent use case for GAMs proposed in [6] and has certain advantages over classical techniques such as Cox regression and Kaplan–Meier estimates, such as the possibility to describe the hazard function while at the same time consider non-linear effects of additional covariates via the use of splines. A key benefit of using the GAM modeling framework for both longitudinal [7] and durability analyses is that it allows for direct comparison of the effects of different covariates across the models.

GAMs produce statistically accurate *p*-values if the number of spline knots (which controls the flexibility of each non-linear effect) is set in advance to a fixed value (in our case, this was 8 for main effects and 4 for interaction terms). Additionally, effect sizes can be read off a partial dependence plot which, for the longitudinal remission model, captures the effect on the log-odds of remission (on the *y*-axis) for changes in the underlying independent variable (*x*-axis). These plots and respective confidence bands were also used to guide variable discretization. Statistical analysis was performed in Python 3.0 using the PyGAM package (0.8.0) and Matplotlib 3.3.3 for plotting purposes.

## 3. Results

### 3.1. Performance of Models

**Figure 2:**
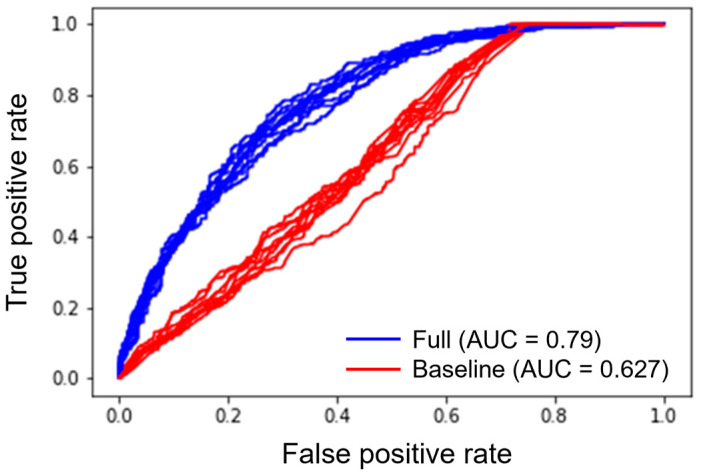
Ten iterations of a training-test split following which the longitudinal remission model is used to classify whether a patient will be in remission at a given week that was not observed in the training data. The full model makes use of all 22 variables (including non-significant ones), whereas the baseline model only uses time.

Performance of the longitudinal remission model in terms of cross-validated R^2^ was modest, around 0.25, though, as discussed, a more reliable metric of predictive performance in this case is AUC, where the model performed well with a value of 0.796 (0.78, 0.8) over 10 folds of cross-validation, compared to that of a baseline model which only considered *time* as a predictor variable, whose median AUC was 0.62 (0.61–0.64) (Figure 2). The performance is comparable with similar analyses such as [8], though the latter additionally used laboratory values shortly after onset of treatment, rather than only at baseline as conducted here.

Given that information about the number of concomitant steroid courses is not available at the time of assessing a patient’s risk of not achieving remission, we reran the analysis without this variable in order to assess the sensitivity of the model to this variable, and the AUC dropped by less than 1%, from a median of 0.796 to one of 0.789.

### 3.2. Significant Variables

The longitudinal remission model considered 22 variables out of which 20 were baseline features and 2 were not (*days since start of UST treatment and number of concomitant steroid courses*). Out of the baseline patient characteristics, 16 proved significant in the non-discretized longitudinal remission model (Table 2). The discretized longitudinal remission model has by construction lower power and was fitted as an interpretability aid; it was performed only on those features that were already found significant from the non-discretized version of the primary analysis model. Most variables preserved their significance, but some lost it, reflecting the importance of discretizing numeric variables such as laboratory measurements. Reassuringly, the shapes, directions, and magnitudes of all reported effect sizes were preserved when discretizing, with the exception of *baseline albumin* and *baseline hemoglobin* which in the non-discretized version seem to have non-linearities not sufficiently captured by the discretized versions, which likely is the reason why these features ceased to be significant in the discretized model. Indeed, several variables lose significance because of discretization, which is expected as discretization loses both information and power.

A number of interaction terms were also included, as shown in Table A1. The drug durability model considered the same baseline variables and given its smaller effective sample size detected seven significant variables, all of which were reassuringly also significant in the remission model.

Variables that were statistically significant across both analyses (longitudinal remission and drug durability) are indicated in bold in Table 2, and comprise the *number of concomitant steroid courses* and the following baseline characteristics, *number of anti-TNF episodes, number of anti-integrin episodes, BMI, age at the time of signing consent, baseline fecal calprotectin, baseline hemoglobin, and baseline CRP*.

The estimated effect of each variable on longitudinal remission is summarized in Table 3. The direction of effect is given for statistically significant variables. The second column describes the directionality of the effect in the non-discretized model. In the third column, the exact effect is reported from the respective feature in the discretized version of the model, in a log-odds scale, as is typical in logistic regressions, including the non-linear version used here (see Appendix A). A positive estimated effect suggests that a higher value of this feature is a positive factor for remission, whereas a negative estimated effect suggests that a higher value correlates with less probability of remission. For factors with more than two values, we report the difference in log-odds of remission between patients in the group with the highest value for this particular feature versus those in the group with the lowest value (holding other things constant).

### 3.3. Position of Ustekinumab in the Treatment Journey

**Figure 3:**
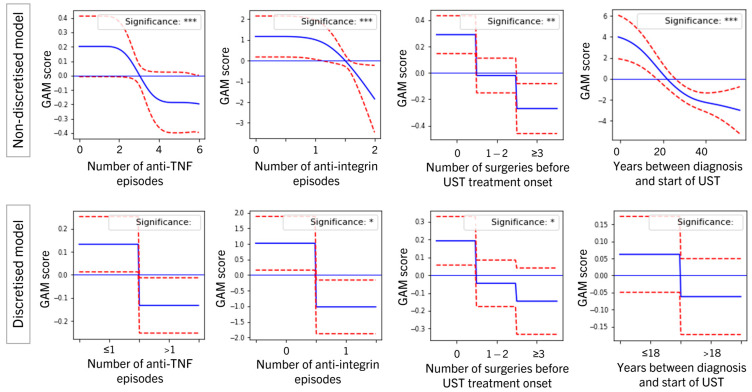
Generalized additive model (GAM) plots showing estimated effects of selected significant variables in the longitudinal remission model, in the non-discretized model (**top row**) and discretized model (**bottom row**). Plots may be read by comparing one value on the *x*-axis to another and can be read off as the vertical distance between the respective *y*-values. Horizontal blue line denotates 0 (no effect), solid blue line represents the estimated effect of different values of the covariates and discontinuous red lines represent 95% confidence intervals. * *p* < 0.05, ** *p* < 0.01, *** *p* < 0.001.

Previous exposure to biologics correlated with lower chances of remission and drug durability. *The number of anti-TNF episodes and number of anti-integrin episodes* were both independent risk factors for lower chances of remission and drug durability, with prior exposure to anti-integrin having a larger effect (Table 3 and Table A1). Although the *number of anti-TNF episodes* was a significant predictor in their continuous form for both remission and durability, the remission discretized version loses significance but maintains a downward trend (Figure 3). Exposure to anti-integrin remains significant in all models, with exposure having a negative effect on the log-odds of remission of up to −1.26 (−2.27, −0.25) compared with a positive effect of no exposure of 0.7 (0.19, 1.22). This ranks it among the variables with the biggest absolute effect in the remission model, with only *BMI at baseline and baseline HBI* ranking higher (see second column in Table 3). These effects persisted in significance and a trend under different ways of capturing exposure, such as replacing the number of episodes with total *cumulative length of episodes* and *exposure* (yes/no).

Additionally, all other variables capturing the position of ustekinumab in the treatment journey (*years between diagnosis and start of UST and number of surgeries before UST treatment onset*) showed a similar correlation with increased odds of remission with earlier positioning (Figure 3 and Table 3).

### 3.4. Role of Concomitant Steroids

**Figure 4:**
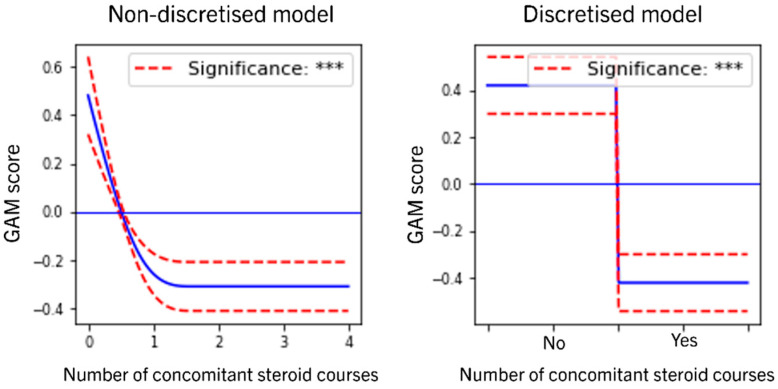
Visual summary of the effect of the number of concomitant steroid courses in the longitudinal remission model, non-discretized model (**left**) and discretized model (**right**). Horizontal blue line denotates 0 (no effect), solid blue line represents the estimated effect of different values of the covariates and discontinuous red lines represent 95% confidence intervals. *** *p* < 0.001.

The *number of concomitant steroid courses* was found to have a significantly negative effect on both remission and durability (Table 2 and Table 3); see the graph in Figure 4 for both longitudinal remission models. A positive association would have suggested that perhaps the observed real-world effect of remission under UST is at least in part due to concomitant steroids. An absent or negative association is consistent with the hypothesis that concomitant steroids are not improving the chances of remission but are instead used in a small subset of most refractory, severe patients who are not responding to treatment to temporarily/modestly alleviating symptoms. This, together with the fact that 57% of the cohort did not receive any concomitant steroids, could be seen as consistent with the hypothesis that a majority of patients experiences steroid-free remission.

## 4. Discussion

This analysis identified 12 baseline factors as negative predictors for achieving clinical remission over time and 4 positive predictors, even after controlling for possible confounders such as disease activity at onset and frailty as proxied by the HBI at baseline and the number of comorbidities, respectively. Baseline variables with a negative effect were *HBI at the first UST dose, years between diagnosis and start of UST, number of surgeries before UST treatment onset number of anti-TNF episodes, number of anti-integrin episodes, age at the time of signing consent, BMI at baseline, number of comorbidities, perianal disease, baseline albumin, baseline fecal calprotectin,* and being *under Immunosuppressants at first UST dose.* On the other hand, *no ileal involvement*, *baseline hemoglobin*, *baseline CRP*, and not being *under steroids at first UST dose* were associated with higher chances of achieving remission in the non-discretized model of longitudinal remission.

Seven variables were also found to be significant independent risk factors across both remission and durability, namely *years between diagnosis and start of UST*, *BMI at baseline*, *baseline fecal calprotectin*, *baseline hemoglobin,* and *baseline CRP*, as well as the *number of anti-TNF episodes* and *number of anti-integrin episodes* the patient was exposed to before the start of treatment, even after controlling for the HBI at baseline and number of comorbidities. Other patient journey characteristics such as the *number of prior surgeries* and the therapeutic latency, as well as disease characteristics such as the location of CD, were statistically significant in the remission model, but not in the durability model (it was expected that the latter would discover fewer findings due to it having less power).

In retrospective non-randomized data such as this cohort [4,9,10,11,12], prior treatment history correlates with disease severity and/or frailty which can therefore act as an unobserved confounder that would explain this finding. Nevertheless, disease severity at onset was controlled for using multivariable analysis involving disease characteristics, baseline HBI values, and laboratory tests, so the fact that this effect persists is conducive to the generation of a hypothesis that earlier positioning of UST in the treatment pathway might be beneficial for patients. It should be noted that this is a single-arm study, and therefore no direct comparison to other treatments is possible.

With regards to baseline laboratory measurements, the study demonstrated that baseline albumin, CRP, hemoglobin, and fecal calprotectin were simultaneously statistically significant predictors of remission, and all except albumin were also statistically significant predictors of durability—while still controlling for disease activity as described above. Higher values of baseline albumin and calprotectin correlated with lower odds of achieving remission, in accordance with others, whereas higher values of baseline CRP and hemoglobin correlated with higher chances of remission and durability. The results for CRP are surprising and in contrast with other reports [12]; given that the model is extensively controlling for disease severity at onset, a hypothesis that would be consistent with this data is that, among severe patients, those with high CRP can be most helped by UST. In particular, the effect size of fecal calprotectin was moderately sized, which confirms clinical practice. This is one of the first analyses also to consider BMI which is often excluded due to missing data considerations and showed it to be a significant independent risk factor for not achieving remission, with one of the largest effect sizes across all baseline covariates for remission. Finally, the location of CD (ileal involvement) and presence of perianal disease were found to be significant as independent risk factors of failing to achieve remission, but insignificant as a predictor of drug durability.

The conventional analysis of this same cohort [4] found three factors associated with ustekinumab drug durability; previous abdominal surgery and concomitant steroid use correlated with drug discontinuation, whereas a maintenance schedule with ustekinumab every 8 or 12 week correlated with drug durability. The differences between the two analyses, conventional multivariate analysis vs. interpretable machine learning, highlight how this novel tool can aid in data-driven discovery of non-linear effects between each predictor and the analyzed outcome.

This report introduces the use of GAMs for flexible interpretable modelling of multiple risk factors in IBD. The additive nature of the model retains an interpretation which is very similar to a logistic regression, and therefore can be couched in language, which is familiar and well understood to the clinician, such as log-odds, but the non-linearity improves the predictive ability of these models for variables that take values over large intervals, such as some laboratory measurements and patient demographics. Traditionally, this is resolved by discretizing the values using clinical intuition. Any form of discretization significantly decreases the statistical power of the model. Expert-based discretization, though helpful for explicability, can suffer in multivariable models where multiple baseline risk factors are controlled for simultaneously, rendering standard thresholds less relevant: for example, after controlling for the BMI and HBI at baseline, the threshold above which a third variable adds extra risk of poor outcomes might differ, as opposed to when it is being considered in isolation. In this work, we used the GAM’s estimated non-linear effects to ascertain the exact threshold at which a variable’s contribution crosses from subtracting risk or being neutral to adding risk, holding everything else constant. The findings between the original form of the model and its discretized version stayed directionally the same, which is reassuring, though the number of statistically significant findings for the latter model fell somewhat, as was also expected.

## 5. Conclusions

Predictors of ustekinumab effectiveness and durability were identified using a cutting-edge approach based on interpretable machine learning and relying on a non-linear multivariable model to control for confounders and identify independent risk factors, which increases the robustness of individual insights. The non-linearity of the model allows for capturing complex effects of time on remission over time, and hence makes use of all follow-up observations rather than selecting a single cross-section, increasing the power of the analysis. It also offers more flexible confounder control, which can increase statistical stability and robustness. Baseline risk for poor outcomes with UST is shown in this analysis to be multifactorial, involving multiple additive risk factors, with no single factor sufficiently explaining poor response by itself, but a combination of several achieving good performance both in terms of statistical goodness-of-fit as well as classification accuracy. In particular, after controlling carefully for disease severity, it was shown that prior exposure to anti-integrins, or higher prior exposure to anti-TNFs, is correlated with worse outcomes, suggesting that earlier positioning of UST might produce better outcomes, though this finding would have to be validated in a randomized trial.

## Figures and Tables

**Table 1:** Variables employed in their original form and discretized.

Variable	Original (Non-Discretized)	Post-Processed (Discretized)	Description of Discretized Version	Descriptives
Days since the start of UST treatment (longitudinal, not baseline)	Numeric	Numeric	-	
HBI at baseline, categorized	Ordinal (3)	-	[5–7] (mild), [8–16] (moderate), >16 (severe)	Distribution: Mild 50%, Moderate 45%, Severe 5% % missing values: 0%
Years between diagnosis and start of UST	Numeric	Dichotomous	≤18, >18	Median: 10.0 % missing values: 0%
Number of surgeries before UST treatment onset	Numeric	Ordinal (3)	0, 1–2, ≥3	Distribution: 0 (47%), 1–2 (43%), ≥3 (10%)
Number of anti-TNF episodes	Numeric	Dichotomous	≤1, >1	Median: 2.0 96.1% of patients ≥1 anti-TNF
Number of anti-integrin episodes	Numeric	Dichotomous	0, 1	Median: 0.0 23.7% of patients ≥1 anti-integrin
Age at the time of signing consent	Numeric	Dichotomous	≤40, >40	Median: 46.0 % missing values: 0%
BMI at baseline	Numeric	Ordinal (3)	<27, 27–38, >38	Median: 23.81 % missing values: 16%
Sex	Dichotomous	Dichotomous	Female, Male	Distribution: Female 49%, Male 51% % missing values: 0%
Number of comorbidities	Numeric	Ordinal (4)	0–1, 2–3, ≥4	Median: 1.0
Perianal disease	Categorical	Dichotomous	Never/Previous, Current	Distribution: Never/Previous 86%, Current 14% % missing values: 0%
CD location	Categorical	Dichotomous	Ileocolic/Ileum (L1/L3), Colon (L2)	Distribution: Ileocolic/Ileum 88%, Colon 12% % missing values: 0%
Patient ever had EIMs	Dichotomous	Dichotomous	Yes/No	Distribution: No 59%, Yes 41%
Family history of CD	Dichotomous	Dichotomous	Yes/No	Distribution: No 13%, Yes 87% % missing values: 16%
Upper gastrointenstinal tract (L4) involved	Dichotomous	Dichotomous	Yes/No	Distribution: No 92%, Yes 8% % missing values: 0%
Baseline albumin (g/L)	Numeric	Dichotomous	≤3.8, >3.8	Median: 4.0 % missing values: 33%
Baseline faecal calprotectin (mcg/g)	Numeric	Ordinal (3)	≤80, 81–650, >650	Median: 667.33 % missing values: 52%
Baseline haemoglobin (g/L)	Numeric	Dichotomous	≤10.9, >10.9	Median: 13.2 % missing values: 10%
Baseline CRP (mg/L)	Numeric	Ordinal (3)	≤0.5, 0.6–6.1, >6.1	Median: 7.2 % missing values: 10%
Steroid use at first UST dose	Dichotomous	Dichotomous	Yes/No	Distribution: No 72%, Yes 28%
Under Immunosuppressants at first UST dose	Dichotomous	Dichotomous	Yes/No	Distribution: No 70%, Yes 30%
Number of concomitant steroid courses	Numeric	Dichotomous	Yes/No	Median: 0.0

BMI, Body Mass Index; CD, Crohn’s Disease; CRP, C-reactive protein; EIM, extraintestinal manifestation; HBI, Harvey Bradshaw Index; TNF, Tumor Necrosis Factor; UST, ustekinumab.

**Table 2:** Statistical significance (*p*-value) of the effect of each variable in longitudinal remission and durability.

	Longitudinal Remission		Durability
Feature	Non-Discretized	Discretized *	
Days since start of UST treatment	**<0.05**	**<0.05**	**<0.05**
HBI at the first UST dose, categorized	**<0.05**	**<0.05**	0.083
Years between diagnosis and start of UST	**<0.05**	0.547	0.077
Number of surgeries before UST treatment onset	**<0.05**	**<0.05**	0.24
**Number of anti-TNF episodes**	**<0.05**	**0.09**	**<0.05**
**Number of anti-integrin episodes**	**<0.05**	**<0.05**	**<0.05**
**Age at the time of signing consent**	**<0.05**	**<0.05**	**<0.05**
**BMI at baseline**	**<0.05**	**<0.05**	**<0.05**
Sex	0.888	N/A	0.842
Number of comorbidities	**<0.05**	**<0.05**	0.296
Perianal disease	**<0.05**	**<0.05**	0.541
Location of CD	**<0.05**	**<0.05**	0.137
Patient ever had EIMs	0.806	N/A	0.648
Family history of CD	0.958	N/A	0.998
Upper gastrointestinal tract (L4) involved	0.999	N/A	0.828
Baseline albumin	**<0.05**	0.431	0.357
**Baseline fecal calprotectin**	**<0.05**	0.26	**<0.05**
**Baseline hemoglobin**	**<0.05**	0.77	**<0.05**
**Baseline CRP**	**<0.05**	**<0.05**	**<0.05**
Under Steroids at first UST dose	**<0.05**	**<0.05**	0.237
Under Immunosuppressants at first UST dose	**<0.05**	**<0.05**	0.813
**Number of concomitant Steroid courses**	**<0.05**	**<0.05**	**<0.05**

Variables that were significant in longitudinal remission (non-discretized) and durability models are indicated in bold. * All features discretized, except for “days since start of UST treatment”. BMI, Body Mass Index; CD, Crohn’s Disease; CRP, C-reactive protein; EIM, extraintestinal manifestation; HBI, Harvey Bradshaw Index; TNF, Tumor Necrosis Factor; UST, ustekinumab.

**Table 3:** Estimated effects of each variable on longitudinal remission.

	Direction of Effect in Non-Discretized Longitudinal Remission Model	Exact Effect Size in Discretized Longitudinal Remission Model (Lowest vs. Highest)	Effect for Group with Lowest Value	Effect for Group with Highest Value
HBI at the first UST dose, categorised	Negative	Negative: −2.32	1.09 (0.91, 1.27)	0.14 (−0.04, 0.32)
Years between diagnosis and start of UST	Negative	Not significant, trending negative	0.06 (−0.05, 0.17)	−0.06 (−0.17, 0.05)
Number of surgeries before UST treatment onset	Negative	−0.33	0.19 (0.06, 0.33)	−0.14 (−0.33, 0.05)
Number of anti-TNF episodes	Negative	Borderline insignificant, trending negative	0.11 (−0.01, 0.24)	−0.11 (−0.24, 0.01)
Number of anti-integrin episodes	Negative	Negative: −1.96	0.7 (0.19, 1.22)	−1.26 (−2.27, −0.25)
Age at the time of signing consent	Negative	Negative: −0.31	0.16 (0.06, 0.26)	−0.16 (−0.26, −0.06)
BMI at baseline	Negative	Negative: −2	0.8 (0.52, 1.09)	−1.2 (−1.73, −0.68)
Sex	Insignificant	N/A	N/A	N/A
Number of comorbidities	Negative	Negative: −0.41	0.22 (0.06, 0.38)	−0.19 (−0.44, 0.07)
Perianal disease	Negative	Negative: −0.7	0.35 (0.21, 0.48)	−0.35 (−0.48, −0.21)
Location of CD (no ileal involvement)	Positive	Positive: 0.74	−0.37 (−0.52, −0.23)	0.37 (0.23, 0.52)
Patient ever had EIMs	Insignificant	N/A	N/A	N/A
Family history of CD	Insignificant	N/A	N/A	N/A
Upper gastrointenstinal tract (L4) involved	Insignificant	N/A	N/A	N/A
Baseline albumin	Negative	Negative: −0.13	0.07 (−0.03, 0.16)	−0.07 (−0.16, 0.03)
Baseline faecal calprotectin	Negative	Negative: −0.45	0.28 (0.02, 0.53)	−0.17 (−0.33, −0.0)
Baseline hemoglobin	Positive	Positive: 0.08	−0.04 (−0.19, 0.11)	0.04 (−0.11, 0.19)
Baseline CRP	Positive	Positive: 0.6	−0.32 (−0.53, −0.11)	0.28 (0.14, 0.43)
Under Steroids at first UST dose	Positive	Positive: 0.69	−0.35 (−0.48, −0.22)	0.35 (0.22, 0.48)
Under Immunosuppressants at first UST dose	Negative	Negative: −0.28	0.14 (0.04, 0.23)	−0.14 (−0.23, −0.04)
Number of concomitant Steroid courses	Negative	Negative: −0.83	0.42 (0.29, 0.54)	−0.42 (−0.54, −0.29)

For factors with more than 2 values, the difference in log-odds of remission between patients in the group with the highest value vs. those with the lowest value are reported. BMI, Body Mass Index; CD, Crohn’s Disease; CRP, C-reactive protein; EIM, extraintestinal manifestation; HBI, Harvey Bradshaw Index; TNF, Tumor Necrosis Factor; UST, ustekinumab.

## Data Availability

Data used for this study cannot be made available due to patient confidentiality, but the first publication of this data may be found at https://academic.oup.com/ibdjournal/advance-article/doi/10.1093/ibd/izab357/6528818 (accessed on 28 July 2022).

## Appendix A. Details of Statistical Methodology

### Appendix A.1. Missing Values

Missing values in either height or weight preclude the calculation of BMI, which is the clinically relevant combination of these two features. Without sophisticated missing data imputation [13], the analysis would either focus on weight alone, which is less clinically relevant, or impute the missing values for height and weight without taking into consideration their correlation and their relationship with other factors. To preserve as much structure as possible in this set of features, an advanced imputation technique known as “iterative Bayesian ridge regression” was used, which is a variant of the well-established Expectation-Maximization algorithm for missing data imputation [7] and relies on a small standalone regression model to predict the missing values using a selection of significant other features as input. Sex, age, and phenotype at the start of UST treatment were found to be significant. Laboratory biomarkers (fecal calprotectin, hemoglobin, albumin, and C-reactive protein) were first carefully calibrated to ensure they all used the same units of measurement and were then imputed using the hot-deck K-nearest neighbors imputation as per [14].

### Appendix A.2. Drop-Out in Longitudinal Analysis

Drop-out bias is a well-known challenge in longitudinal analysis. Analyses that have a non-linear estimation shape are more robust to such bias in the sense that it is unlikely to impact the shape of the remission trajectory earlier on in the treatment window but can still be affected at later parts of the trajectory where the prevalence of drop-out increases. In a longitudinal setting, additional timestamps were generated for drop-out patients using the LVCF imputation, so that each of these patients would not have a number of visits smaller than a value randomly drawn from the distribution of the number of doses received by non-drop-out patients.

### Appendix A.3. Shape Constraints

The user can either manually pre-specify the number of splines (which control the smoothness of the function $s_{j}$) or use cross-validation to determine the optimal number instead, in which case, however, the p-values and confidence intervals are no longer valid. For this reason, splines were pre-set to fixed values. All continuous variables were modeled using splines, whereas factors were modeled as in logistic regression as constant effects.

Modern implementations of GAM additionally allow the introduction of shape constraints into the smoothing functions, including monotonically decreasing, monotonically increasing, convex and concave shapes. Monotonicity is a natural generalization of linearity. For each variable, a monotonic decreasing and a monotonic increasing constraint were tried out, and the one resulting in the best fitting model was used, presented in Table 3.

### Appendix A.4. Interaction Effects

In all models, a number of interaction effects were introduced in the model only where the main effects were first found to be significant: these were an interaction between hemoglobin and sex, interactions between each baseline lab and the HBI at the first UST dose, and, for longitudinal remission only, an interaction term between the days since the start of treatment and years since diagnosis, as well as an interaction between days since the start of UST treatment and BMI. These improved the fit of the model without modifying the shape or direction of the main effects.

**Table A1:** Interaction effects.

Feature Interactions	*p*-Value of Effect		
	Longitudinal Remission		Durability
	Numeric Values Used for Several Features	All Features Discretized, Except for “Days since Start of UST Treatment”	
(Baseline hemoglobin) × (Sex)	0.626	N/A	0.821
(HBI at the first UST dose, categorized) × (Baseline fecal calprotectin)	<0.05	N/A	0.307
(HBI at the first UST dose, categorized) × (Baseline albumin)	<0.05	N/A	0.239
(HBI at the first UST dose, categorized) × (Baseline hemoglobin)	<0.05	N/A	0.306
(HBI at the first UST dose, categorized) × (Baseline CRP)	<0.05	N/A	<0.05
(Days since the start of UST treatment) × (Years between diagnosis and start of UST)	<0.05	N/A	N/A
(Days since the start of UST treatment) × (BMI)	<0.05	N/A	N/A
